# Identical chemotherapy schedules given on and off trial protocol in small cell lung cancer response and survival results

**DOI:** 10.1038/sj.bjc.6600433

**Published:** 2002-08-27

**Authors:** J A Burgers, A Arance, L Ashcroft, J Hodgetts, L Lomax, N Thatcher

**Affiliations:** University Hospital Rotterdam, Department of Pulmonary Diseases. P.O. Box 5201, 3008 AE Rotterdam, The Netherlands; Christie Hospital NHS Trust, Department of Medical Oncology, Wilmslow Road, Manchester M20 4BX, UK

**Keywords:** randomised trial, participation, survival

## Abstract

Patients who are treated within clinical trials may have a survival benefit dependent on being a trial participant. A number of factors may produce such beneficial outcome including more rigorous adherence to a peer reviewed trial protocol, management by an experienced treatment team, being treated in a specialist centre etc. The current investigation compared patients treated on and off trial with the same standard arm treatment regimen. The results could then be interpreted without the confounding factors of differing treatment regimens, treatment teams or treatment hospitals. The results demonstrated given these circumstances that survival was no different for patients participating in a randomised trial compared with a group of patients similarly treated who were not eligible for trial entry or who declined randomisation. These results were obtained by the rigorous adherence to a defined protocol with the invaluable assistance of designated lung cancer staff.

*British Journal of Cancer* (2002) **87**, 562–566. doi:10.1038/sj.bjc.6600433 www.bjcancer.com

© 2002 Cancer Research UK

## 

Chemotherapy is the cornerstone for the management of small cell lung cancer (SCLC). One of the chemotherapeutic regimens that has been employed as standard treatment for many years, consists of cyclophosphamide, doxorubicin and etoposide (CDE). Response rates are high (70–90%), but as with other chemotherapeutic regimens recurrence frequently occurs. The resulting median and 2-year survival rates are of the order of 12 months and 20% respectively in patients with good performance status (Souhami and Law, 1990; Thatcher *et al*, 2000). Attempts to enhance the CDE regimen by the addition of cisplatin or alternating CDE with vincristine, carboplatin, ifosphamide combination did not result in any improvement of survival (Postmus *et al*, 1996; Urban *et al*, 1999). However, dose intensification by 2-weekly administration with growth factor support improved the 2-year survival from 8 to 13% compared to the standard 3-weekly CDE schedule, without additional toxicity (Thatcher *et al*, 2000). Adjuvant thoracic irradiation and prophylactic cranial irradiation have also led to a modest survival benefit which did not depend on the initial chemotherapy schedule (Pignon *et al*, 1992; Arriagada *et al*, 1995; Auperin *et al*, 1999).

However, the outcome of SCLC is not solely related to the antineoplastic therapy. Clinical factors including performance status, disease stage, serum sodium, liver function tests, lactate dehydrogenase are important prognostic survival indicators in SCLC. In order to identify patients with differing prognoses and risk of early death, these factors have been combined in scoring systems (Sagman *et al*, 1991; Thatcher *et al*, 1995; Lassen *et al*, 1995).

An intriguing question is whether patients treated within a trial protocol have an inherently better survival than patients who are treated ‘off study’. The finding of statistically significant higher survivals among trial participants than in trial control patients was highlighted in childhood leukaemia (Stiller and Draper, 1989). Subsequently a beneficial survival effect for participants has been noted in myeloma, nephroblastoma, non Hodgkin lymphoma and sarcoma trial patients (Karjalainen and Palva, 1989; Lennox *et al*, 1979; Wagner *et al*, 1995; Antman *et al*, 1985).

The benefit also extended to non-small cell lung cancer where survival advantage was also apparent for the participants in a trial for resectable early stage disease (Davis *et al*, 1985). More recently small cell lung cancer patients have been reported as having a better survival when treated within a study protocol rather than patients treated off protocol (Quoix *et al*, 1986; Schea *et al*, 1995). However, these examples suggesting that protocol treatment is beneficial to the patient refer to situations where the therapy offered on and off protocol were different which could account for the survival difference. The Health and Technology Assessment programme of the NHS reported on some of the issues of randomised clinical trials (RCTs), including the effects of participation (Edwards *et al*, 1998). Assessment was made of the trial effects by plotting the hazard ratios derived from the data set and led to a view that ‘RCTs tend to be good for you if there is a pre-existing effective treatment that is included in the trial protocol or if it turns out that the experimental treatment is more effective.’ However, even without bias, any survival benefit for trial participants may be due to the effect of a particularly successful intervention within the trial or ipso facto being a part of the trial itself (Edwards *et al*, 1998). Nevertheless the comparison of trial results with those ‘off trial’ but using the same treatment protocol does not seem to have been addressed. To try and dissect the issue further we compared the demographics, treatment characteristics and survival data of patients with SCLC who were treated on and off trial but with an identical chemotherapy regimen, the same treatment guidelines, similar patient characteristics and by one treatment team.

## MATERIALS AND METHODS

The data of patients with SCLC who were treated with CDE chemotherapy at the Christie Hospital between 1 January 1994 and 1 March 1998 were reviewed retrospectively. During this period patients were recruited into two phase III randomised clinical trials which investigated the use of CDE chemotherapy as the standard treatment arm. One trial has been published (Thatcher *et al*, 2000) and the other comparing VICE (vincristine, ifosfamide, carboplatin and etoposide) against a standard regimen which included CDE has completed recruitment but not yet published. The inclusion criteria for both trials were similar, i.e. previously untreated patients with a good prognostic score, adequate liver, renal function and blood counts (Thatcher *et al*, 2000). Patients could have extensive stage disease providing the other prognostic factors which included biochemical indices and performance status were not adverse (Cerny *et al*, 1987). All eligible patients were invited to participate in one of the two clinical trials. If this for any reason was not possible due to patients declining, logistics etc. the off study chemotherapy that was offered during this time was the CDE schedule. Both on and off trial, the standard regimen consisted of 40 mg m^−2^ doxorubicin, 1 g m^−2^ cyclophosphamide and 120 mg m^−2^ etoposide i.v. on day 1 and 240 mg m^−2^ on day 2 and 3 was given orally. The cycles were repeated every 3 weeks with a maximum of six cycles or until tumour progression or severe side effects prohibited therapy.

The next cycle of chemotherapy was only given if the total WBC was ⩾3000 μl, the neutrophils count was ⩾1500 μl and the platelet count ⩾100 000 μl. Dose reduction was not recommended and growth factor support was allowed. An identical policy e.g. interval, between cycle blood counts was also applied to the off trial patients. Each patient was assessed pre-treatment, at each attendance for treatment and regularly there after. A full clinical examination, blood counts, biochemistry, chest X-ray and other relevant tests were performed. In case of limited stage disease patients were offered thoracic radiotherapy. Prophylactic cranial irradiation was offered to limited stage patients with a complete tumour response.

Patients for the current analysis were identified from the records of the hospital pharmacy which contained all patients treated with the CDE regimen. Furthermore a double check was performed by a search of the hospital treatment records for the same period to identify all small cell lung cancer patients who received any treatment. The hospital files of all 106 CDE relevant patients could be traced out of a total of 798 patients with small cell lung cancer referred to the hospital. The other patients were either on trials using different regimens or off trial treated with a ‘non-CDE’ regimen. The medical records were then screened for the demographics of the patients, the prognostic factors at the time of diagnosis, the number of chemotherapy cycles given and the treatment delay between the chemotherapy cycles, adjuvant radiotherapy and the survival.

### Statistical analysis

Overall survival curves were produced using the Kaplan–Meier method, and were compared using the log-rank test. The patient characteristics and response rates were assessed using Person's χ^2^ test and Fisher's Exact test.

## RESULTS

One hundred and six SCLC patients were treated with CDE chemotherapy in the Christie Hospital in the period 1 January 1994 until 1 March 1998. Sixty patients were treated within one of the two randomised trials and 46 patients were treated off trial. The characteristics of both patient groups are given in Table 1

**Table 1:**
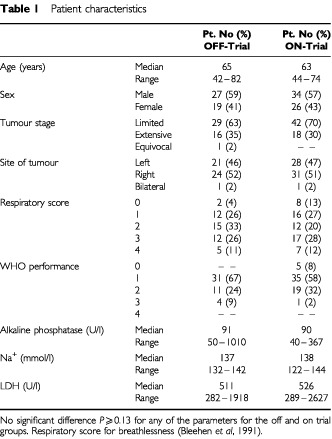
Patient characteristics

. Patients treated on and off trial did not differ significantly in their demographic and prognostic factors. Although the patients treated on trial showed a tendency toward a better performance status, this was not statistically significant.

The treatment given to both patient groups did not differ significantly. The number of cycles of chemotherapy given and the delay between the cycles were comparable. Consolidation radiotherapy of the thorax and prophylactic cranial irradiation was offered to a similar percentage of patients (Table 2

**Table 2:**
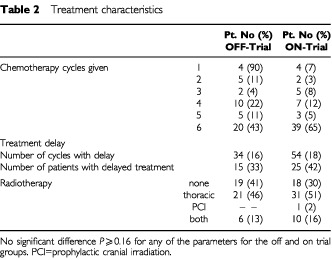
Treatment characteristics

).

For all patients not treated within the trial protocol, the reasons were documented and are given in Table 3

**Table 3:**
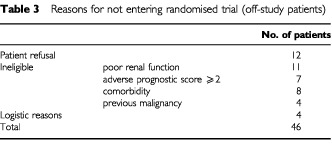
Reasons for not entering randomised trial (off-study patients)

. Only 12 out of 46 patients declined trial entry (reasons were not requested), four patients could not be entered for logistic reasons (holidays etc.) and the other patients were ineligible due to clinical reasons the main being impaired renal function.

The tumour response rates derived from radiological reports of both treatment groups are given in Table 4

**Table 4:**
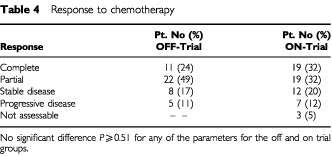
Response to chemotherapy

; again no differences were detected. Furthermore the survival analysis did not reveal any difference between the patients treated on and off study. The median survival for all patients was 345 days (11.3 months), with 330 days (10.9 months) for the patients treated off trial and 346 days (11.4 months) for the patients treated on trial. In both patient groups, two patients were alive without evidence of disease at the time of analysis and in both groups two patients died of treatment related complications. Three patients died from a non-tumour related cause – ischaemic cardiac disease.

## DISCUSSION

Analysis of the survival data of the SCLC patients with CDE chemotherapy did not reveal any difference between the patients' treated within a trial protocol and those outside the trial. The finding is in contrast with reports suggesting that on study treatment is an independent prognostic factor, correlated with better survival (Davis *et al*, 1985; Schea *et al*, 1995; Quoix *et al*, 1986; Wagner *et al*, 1995). However, in these studies the treatment regimens on and off trial protocol were different.

Survival may differ considerably between patients on and off study because of a differing mix of prognostic factors in the patient groups. Indeed few study protocols in SCLC include patients with poorer prognostic factors thereby favouring survival of patients treated within a particular study when compared with a more general population (White *et al*, 2001). However, this difference was not apparent in the current patient population due to consistency in applying a prognostic scoring system to identify patients for particular treatment regimens, i.e. CDE chemotherapy. The system allowed patients with either extensive disease or a poor performance status to be included on trial provided that this was the only adverse prognostic factor. As a result the trial population was reflected in the off study population that was analysed. Furthermore, the distribution of males and females between the two study groups was very similar, minimising the effect of the possible favourable prognostic factor of female gender (Johnson *et al*, 1988). Reports claiming a benefit for the control group treated within a trial have in the past compared different treatment regimens given on and off study, which, could account for any survival difference (Edwards *et al*, 1998). In the case of identical on and off trial therapies, survival could be influenced by the existence of more explicit on study treatment protocols, more rigid adherence to treatment schedules and follow-up guidelines (Colette *et al*, 1999). In the current investigation patients both on and off trial received a comparable number of chemotherapy cycles and had a similar delay in between the cycles. The received dose intensity, and total dose were therefore similar as was the adjuvant thoracic irradiation and prophylactic cerebral irradiation. The study was retrospective, but assuming the off trial patients would do worse, with 106 patients and a 15% 2-year survival there would be a 70% power to detect a 10% difference with a one-tailed test and α=5%. The relatively high number of patients on trial was due in part to the simple trial protocol design. The inclusion criteria were applicable to many patients and the management protocols for handling haematological toxicity and follow up guidelines were a reflection of routine (off study) practice. Studies with laborious protocols or where the treatment arms are very different, e.g. chemotherapy or not, or where there are restrictive patient trial entry criteria tend to have more problems with patient recruitment; as illustrated by the 73% refusal rate of eligible patients in a multi-modality NSCLC trial (Spiro *et al*, 2000). Indeed of all eligible patients only 25% agreed to be randomised despite the known willingness amongst cancer patients to participate in clinical research (Slevin *et al*, 1995). However, other audits, for example of lymphoma, achieved higher recruitment rates of 45%. The main reasons here for ineligibility were medical, no current study being available and differences of histological opinion (Hancock *et al*, 1997). With fewer patients being excluded from study treatment, patient outcomes are more directly applicable to routine practice (Quoix *et al*, 1986). Indeed in our study only 10% of patients who were eligible declined to be randomised. Nevertheless patient selection, either by the patient or physician's choice, is known to influence the distribution of differing tumour stages on and off study (Antman *et al*, 1985; Quoix *et al*, 1986). This discrepancy was not apparent in our audit which involved a written ‘non trial’ protocol and treatment by a single cohesive clinical and specialist nursing group experienced in the daily running of trials. Tight organisation of medical care, specified treatment by protocol, and treatment at specialist centres is known to be of survival benefit (Stiller, 1994).

A number of limitations however, should be kept in mind during interpretation of results of the current study. The design of the study was retrospective and patient numbers limited. Nevertheless data on all patients treated with CDE in the given time period were retrieved and were analysed. It is important to know the reasons for not including patients into a trial protocol since this might give insight into the magnitude of selection of patients (Quoix *et al*, 1986). In our study a relatively high percentage of patients were included in trials. The other SCLC patients were treated in our institution during the time period on different protocols, e.g. those with a better prognostic score were treated in protocols of intensive chemotherapy. The main reasons for not treating patients within trial were medical which accounted for 65% of the patients as can be seen from Table 3, a percentage similar to other SCLC studies (Quoix *et al*, 1986). We have no data on non clinical prognostic factors which could be important such as socio-economic status. Lung cancer patients living in better socio-economic areas are known to have more favourable survival compared to patients living in the lower socio-economic areas of the southeastern part of the Netherlands (Schrijvers *et al*, 1995). This observation was confirmed by the Cancer and Leukaemia Group B, which reported that small cell lung cancer patients with a lower annual income and lower educational level had a shorter survival than those with higher income or education (Cella *et al*, 1991). Indeed in the UK the effects of deprivation on cancer survival have been reported across a variety of adult cancers, including lung (Sharp, 1999). Large differences in survival were noted and for the most affluent patients between 5 and 16% were more likely to be alive after 5 years than the more deprived patients (Anderson, 1999). Other non-disease related factors – the experience of the clinician or institution can influence survival as in poor prognosis non seminoma patients (Collette *et al*, 1999). In this investigation physicians treating patients at institutions with low accrual (<5 patients per annum) may not have felt comfortable with the demanding protocol and the management of toxic effects leading to dose attenuation and failure to adhere to the protocol (Collette *et al*, 1999; Feuer *et al*, 1999). Clearly if the treatment is relatively ineffective in terms of survival outcome, e.g. palliative radiotherapy in advanced NSCLC, then there is unlikely to be major impact even when large patient numbers are treated (Wheeler *et al*, 1994).

While some studies which use matched patient populations and multivariate statistical techniques claim that on study treatment might be an independent prognostic factor, other studies including ours did not confirm this view (Edwards *et al*, 1998). Results of studies, especially phase III trials can be more directly translated to daily practice if the on study patient population mirrors that of the off study population. The current finding lends confidence to the concept that strict adherence to a ‘control’ protocol by an experienced centralised treatment team including dedicated lung cancer nurses does not result in an inferior effect in terms of survival to that obtained if the same treatment regimen within the trial context. Such a process does not substitute for the solid evidence base obtained for randomised clinical trials.
